# Overcoming COVID-19: Addressing the perception of risk and transitioning protective behaviors to habits

**DOI:** 10.1017/ice.2020.284

**Published:** 2020-06-09

**Authors:** Mohamad G. Fakih, Lisa K. Sturm, Rand R. Fakih

**Affiliations:** 1 Quality & Clinical Integration, Clinical & Network Services, Ascension, St Louis, Missouri; 2 Wayne State University School of Medicine, Detroit, Michigan; 3 Wayne State University, College of Education, Detroit, Michigan

*To the Editor*—We started this year facing an incredible challenge that defied many assumptions regarding pathogens. The coronavirus disease 2019 (COVID-19) has spread from China across borders and oceans to become a pandemic. Our unfamiliarity with the nature of the disease, from asymptomatic infection to presymptomatic contagiousness, to its efficiency of transmission and the varied nonspecific clinical presentations, made containment of its early spread difficult.

The risk of contracting COVID-19 infection depends on the prevalence within a community, the efficiency of viral transmission, and the behavior of the susceptible host. Some regions within the United States have been disproportionately affected,^1^ but the uncertainty related to the risk for exposure and severity of outcome, combined with the desire to return to our normal lives for economic and mental health reasons, has led to varied practices across the nation. Social distancing (shelter in place or stay at home) imposed by states, curtailed the outbreak in many of our communities. In the absence of a treatment or a vaccine that delivers protection, along with greater herd immunity, effective management of this disease is contingent to a large extent on the protective behaviors adopted by the population at large.

For decades, we have struggled with hardwiring the compliance with hand hygiene before and after caring for patients, although many providers acknowledge that poor compliance leads to transmission of multidrug-resistant organisms and patient harm.^2^ We also accept contracting a cold or influenza as an unavoidable, expected event. We marginally altered our behavior even when a seasonal influenza vaccine was not a good match. The contrast between our approach to COVID-19 and previous epidemics and pandemics is our perception of risk. The mortality of COVID-19 patients is thought to be up to 10 times that of influenza, creating the urgency to intervene.^3,4^ On the other hand, other threats to society, such as climate change are uncommonly perceived as an emergency, hence the action may not be pressing. Our reaction to COVID-19 threat has been based on our variable interpretation of such risk.

The approach to curbing further transmission of COVID-19 within communities focuses on the institution of measures (1) to detect and isolate those infected, (2) to practice point source control, (3) to reduce environmental contamination, and (4) to optimize engineering controls. Symptoms and epidemiologic risk (eg, travel, known exposure) screening has been widely used to identify suspected cases as a prequel to molecular testing and identifying active infection. Testing, however, is not failure proof and may risk providing a false sense of security. Recent data on rapid polymerase chain reaction testing reported potentially inaccurate results.^5^ On the other hand, instituting behaviors such as self-isolation for 10–14 days prior to a surgery, eliminates the risk of a patient being actively infected at the time of the procedure. Environmental cleaning reduces the chance for persons to contact contaminated surfaces, and engineering control through deploying spatial separation and reducing crowding will lessen the chances of exposure to the pathogen. To achieve long-lasting benefits that help reduce COVID-19 and other future infectious outbreaks, we will need to learn and adopt precautionary actions.

One of the main predictors of people’s engagement in protective behavior is risk perception.^6-8^ People are more likely to comply with the recommended precautionary behaviors if they think that they are susceptible to contracting the disease (ie, perceived vulnerability) and if that illness is deemed to lead to severe health consequences (ie, perceived severity).^7,9^ However, according to the protection motivation theory,^7^ risk perception is an imperative but insufficient precursor for the adoption of protective behaviors.^6^ In times of stress, people also assess their ability to cope with the threat (ie, coping appraisal). Thus, the motivation to engage in protective behavior is heightened with the belief that the recommended protective measures would lead to successful outcomes (ie, response efficacy) and when there is confidence in one’s ability to perform healthy behaviors (ie, self-efficacy; for example, “I am able to constantly perform hand hygiene and mask.”).^7^ In addition, risk perception and self-efficacy beliefs are greatly influenced by the sources of information to which individuals are exposed (eg, media, friends, governmental health agencies). Information deemed questionable or coming from a noncredible source may negatively influence one’s motivation to adopt protective behavior.^6^

Behavioral change requires understanding the risk we are facing, learning through effective means how to mitigate the risk, and sustaining protective behaviors to become effortless habits.^10^ We suggest moving our population to adopt healthy behavior as the primary venue to minimize exposure to pathogens transmitted through droplets or contact. This includes regular hand hygiene, no hand shaking or sharing objects, following respiratory etiquette, and avoiding exposure to those that are sick. In addition, we need to continue public education on how to perform hand hygiene, how to mitigate the risk of fomite transmission, and how to use cloth face coverings correctly. The messages are then culturally accepted and espoused by the community so the actions become part of our regular activities. In addition, structural changes within the community and public settings may be required to establish an environment that is favorable to the concepts of minimizing ongoing risk. Finally, similar to enjoying our coffee every morning as part of our daily routine, we will need to experience the satisfaction and reward of performing these safe behaviors as an anchored habit.
